# Transcriptional profiling of macrophages reveals distinct parasite stage-driven signatures during early infection by *Leishmania donovani*

**DOI:** 10.1038/s41598-022-10317-6

**Published:** 2022-04-16

**Authors:** Visnu Chaparro, Tyson E. Graber, Tommy Alain, Maritza Jaramillo

**Affiliations:** 1 Institut National de la Recherche Scientifique (INRS) – Centre Armand-Frappier Santé Biotechnologie (AFSB), 531 boul. des Prairies, Laval, Québec H7V 1B7 Canada; 2 Children’s Hospital of Eastern Ontario Research Institute, Ottawa, ON Canada; 3 Department of Biochemistry, Microbiology and Immunology, University of Ottawa, Ottawa, ON Canada

**Keywords:** Parasitology, Transcriptomics, Gene regulation in immune cells, Immune evasion, Infection

## Abstract

Macrophages undergo swift changes in mRNA abundance upon pathogen invasion. Herein we describe early remodelling of the macrophage transcriptome during infection by amastigotes or promastigotes of *Leishmania donovani*. Approximately 10–16% of host mRNAs were differentially modulated in *L. donovani*-infected macrophages when compared to uninfected controls. This response was partially stage-specific as a third of changes in mRNA abundance were either exclusively driven by one of the parasite forms or significantly different between them. Gene ontology analyses identified categories associated with immune functions (e.g. antigen presentation and leukocyte activation) among significantly downregulated mRNAs during amastigote infection while cytoprotective-related categories (e.g. DNA repair and apoptosis inhibition) were enriched in upregulated transcripts. Interestingly a combination of upregulated (e.g. cellular response to IFNβ) and repressed (e.g. leukocyte activation, chemotaxis) immune-related transcripts were overrepresented in the promastigote-infected dataset. In addition, Ingenuity Pathway Analysis (IPA) associated specific mRNA subsets with a number of upstream transcriptional regulators predicted to be modulated in macrophages infected with *L. donovani* amastigotes (e.g. STAT1 inhibition) or promastigotes (e.g. NRF2, IRF3, and IRF7 activation). Overall, our results indicate that early parasite stage-driven transcriptional remodelling in macrophages contributes to orchestrate both protective and deleterious host cell responses during *L. donovani* infection.

## Introduction

Macrophages are the main replicative niche of protozoan parasites of the genus *Leishmania*, the etiologic agents of a spectrum of vector-borne diseases known as leishmaniases^1^. Within macrophages, sandfly-transmitted *Leishmania* promastigotes transform into amastigotes while subverting numerous host cell processes and immunological functions to ensure their proliferation^1^. Visceral leishmaniasis (VL) is a life-threatening disease that is caused by *L. donovani* and *L. infantum* (syn. *L. chagasi*)^2^. VL is endemic in more than 60 countries where it represents a severe public health concern due to the lack of vaccines and the emergence of parasite drug resistance^3^. Hence, a better understanding of the molecular events occurring at the host cell–parasite interface is critical to identify novel regulatory nodes for therapeutic intervention.

Transcriptomic studies of macrophages infected with promastigotes of different *Leishmania* spp. (*L. major*, *L. amazonensis*, *L. chagasi*) indicate that the most distinctive changes occur in early stages after parasite internalization (i.e. 1–12 h post-infection)^4–7^. Even though *L. donovani* promastigotes elicit the activation of anti-parasitic intracellular signals in macrophages as early as 15 min post-infection^8^, they are able to dampen host cell responses involved in pathogen clearance within 6 h (e.g. phagolysosome maturation, antigen presentation, oxidative burst, and apoptosis)^9–12^. Consistent with this, rapid modulation of multiple transcription factors (e.g. STAT1, NRF2, IRF3 and IRF7) has been associated with either parasite persistence or host cell defense mechanisms against *L. donovani*^12–15^. The first host cell gene expression profiles were performed in human and mouse primary macrophages infected with *L. donovani* amastigotes^16^ or promastigotes^17,18^ for 16 h to 96 h using DNA microarrays. However, this technique has several limitations (e.g. hybridization issues, limited probe availability, lower detection of splice junctions and rare or novel transcripts, etc.)^19^. Subsequent transcriptional signatures of macrophages infected with *L. donovani* promastigotes were defined using RNAseq^20,21^, which outperforms earlier technologies by allowing transcriptome-wide direct sequencing^19^. Two recent RNAseq-based studies carried out in murine macrophages infected with *L. donovani* promastigotes reported rapid changes in abundance of transcripts associated with host cell lipid and glutamine metabolic activity (6 h post-infection)^22,23^. Intriguingly, the global transcriptional response of macrophages to early infection was not analyzed in depth^22,23^.

In all, currently available datasets may not reflect the totality of changes in gene expression programs that trigger, or are elicited by, early macrophage responses during *L. donovani* infection. Of note, to the best of our knowledge, no high throughput comparative study of early transcriptional changes in macrophages driven by both stages of *L. donovani* is available to date. Herein, using RNAseq datasets from mouse primary macrophages infected with *L. donovani* amastigotes and promastigotes for 6 h, we describe broad yet selective changes in the transcriptome of the host cell that are likely to tailor key early cellular responses involved in host defense but also in disease progression during VL.

## Results

### Infection with *L. donovani* amastigotes or promastigotes promotes early changes in the mRNA pool of the host cell

**Figure 1:**
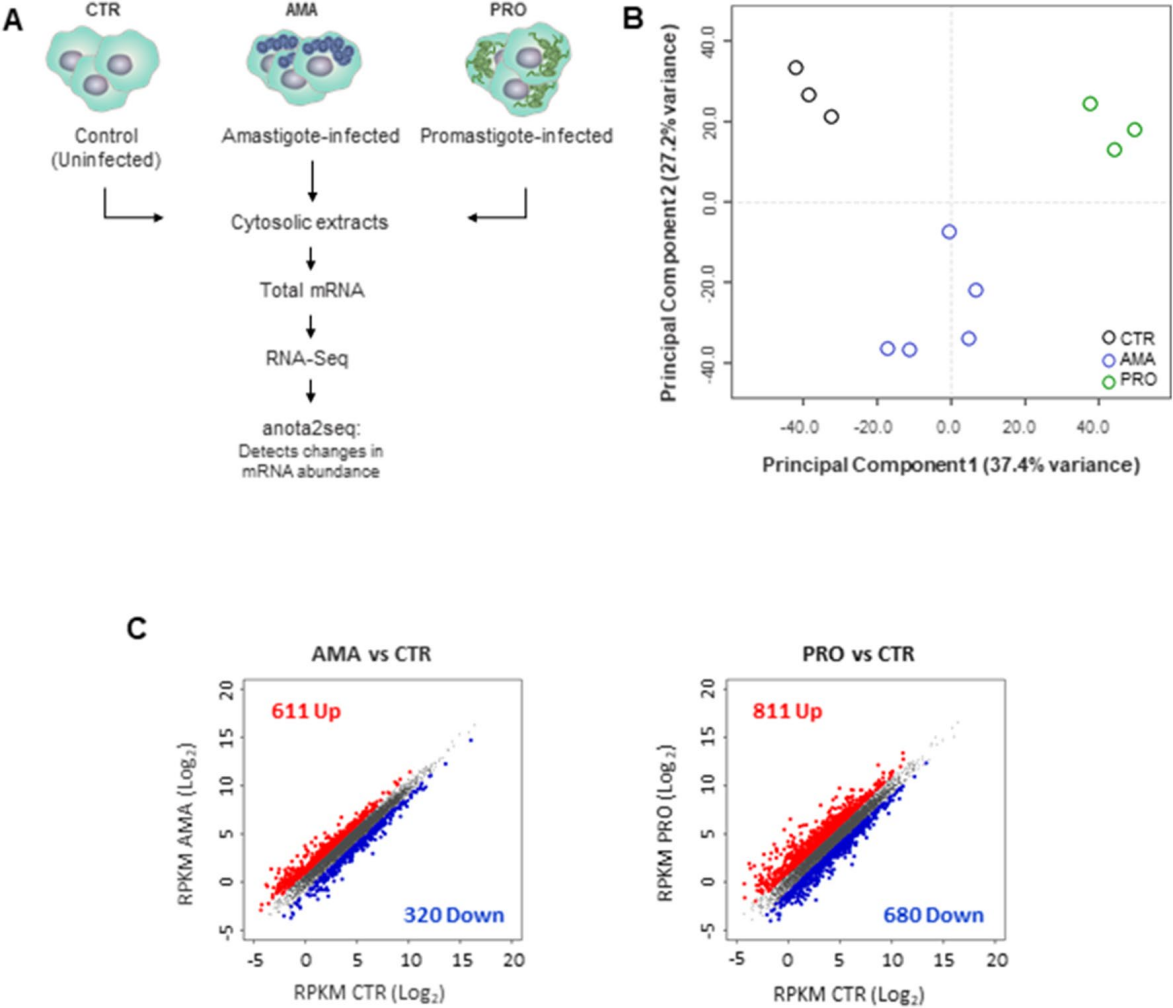
*L. donovani* infection promotes early transcriptome-wide changes in macrophage mRNA abundance. (**A**) Strategy to identify cytosolic mRNAs that are regulated in *L. donovani* amastigote (AMA)- or promastigote (PRO)-infected BMDMs. RNAseq experiments were carried out in three to five biological replicates per condition. (**B**) Cytosolic mRNA datasets of BMDMs infected or not with *L. donovani* AMA or PRO were projected on the first two components of a principal component analysis. (**C**) Scatter plots of gene expression as RPKM (log_2_) values for total cytosolic mRNA. Differentially regulated transcripts are indicated in red (upregulated) or blue (downregulated). Unchanged mRNAs are shown in grey.

To compare the early effects of the two life stages *L. donovani* in the mature mRNA pool of the host cell, total cytosolic mRNA extracts from bone marrow-derived macrophage (BMDM) cultures infected with amastigote (AMA) or promastigote (PRO) parasites for 6 h were subjected to RNAseq and compared to non-infected controls (CTR) (Fig. 1A). As shown by a projection of a principal component analysis, infection appears to be the main source of variation (PC1 = 37.4%) between the different datasets followed by a distinctive distribution of AMA samples along the second component (PC2 = 27.2%) (Fig. 1B). Differentially regulated mRNAs were identified using the anota2seq algorithm with a false discovery rate (FDR) ≤ 0.05 and a log_2_ expression fold-change ≥ 1.0. Out of 9442 mRNAs detected in BMDMs, 9.9% showed differential abundance during *L. donovani* amastigote infection (65.6% upregulated and 34.4% downregulated) (Fig. 1C left panel and Table S1) while 15.8% were altered in BMDMs following infection with the promastigote stage (54.4% upregulated and 45.6% downregulated) (Fig. 1C right panel and Table S1). These data indicate that infection by either amastigotes or promastigotes of *L. donovani* leads to early reprograming of the mRNA content of the host cell.

### Early transcriptional changes in macrophages infected with *L. donovani* amastigotes are associated with the inhibition of cell death and immune functions

**Figure 2:**
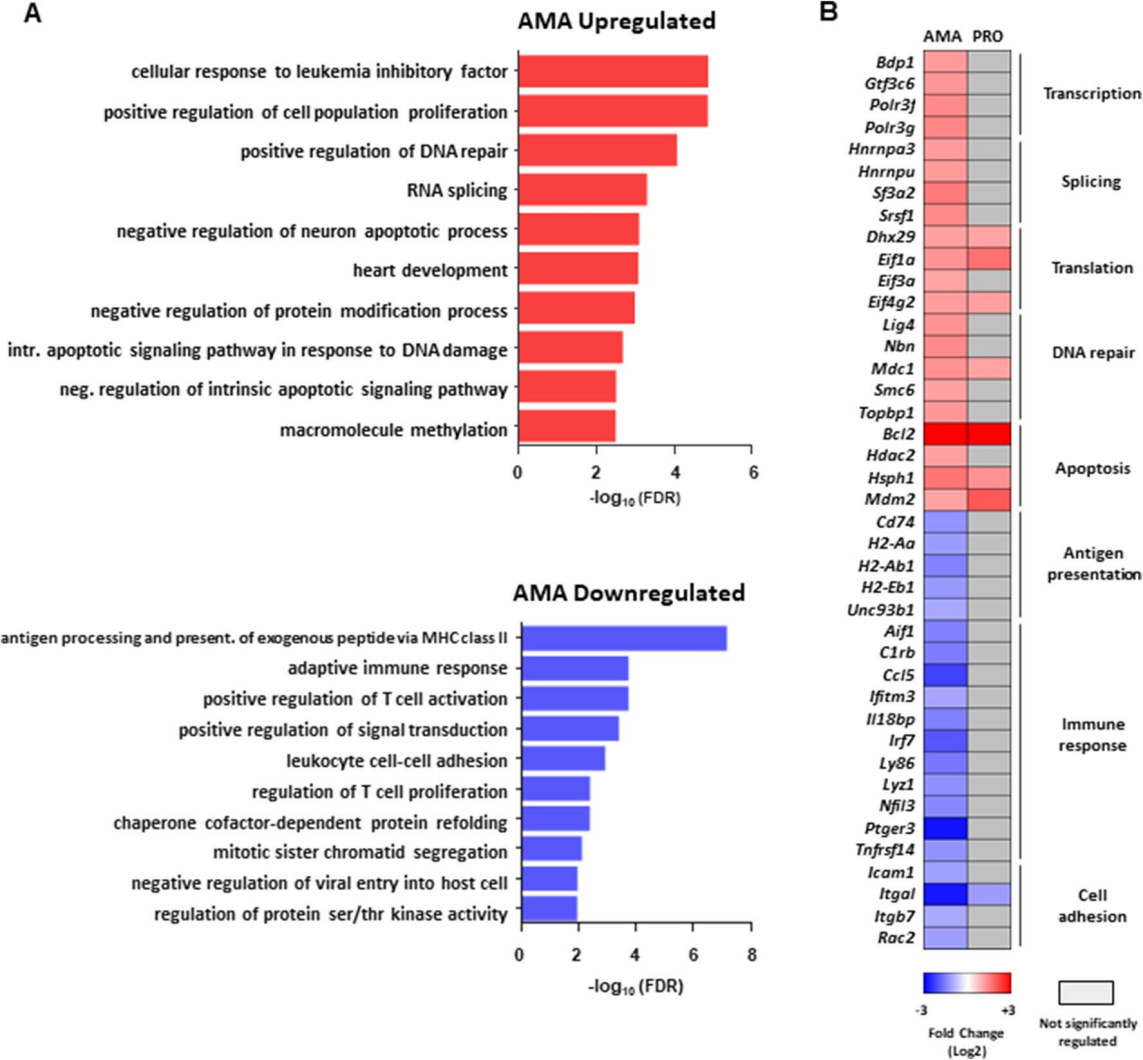
Selective changes in mRNA abundance predict amastigote-specific modulation of cell death and immune functions in macrophages during *L. donovani* infection. (**A**) FDR values (− log_10_) for selected GO term enriched categories of differentially up- or downregulated mRNAs upon *L. donovani* AMA infection. (**B**) Changes in mRNA abundance for selected genes in enriched GO terms. Analyses were carried out on data generated from at least three biological replicates.

Gene Ontology (GO) hierarchical clustering analysis was carried out to determine whether subsets of mRNAs encoding functionally related proteins are selectively modulated in BMDMs upon infection with *L. donovani* amastigotes (Fig. 2A and Table S2). Enrichment of functional categories related to regulation of gene expression, positive regulation of DNA repair, and negative regulation of apoptosis and protein modification was detected in the AMA-upregulated dataset (Fig. 2A upper panel, and Table S2). Targets in these categories included transcripts that encode transcription (*Bdp1*, *Gtf3c6*, *Polr3f.*, *Polr3g*), splicing (*Hnrnpa3*, *Hnrnpu*, *Sf3a2*, *Srsf1*) and translation (*Dhx29*, *Eif1a*, *Eif3a*, *Eif4g2*) factors, proteins involved in DNA repair (*Lig4*, *Mdc1*, *Nbn, Smc6*, *Topbp1*), and inhibitors of apoptosis (*Hdac2*, *Hsph1*, *Mdm2*) including *Bcl2* which was highly upregulated by both parasite stages (Fig. 2B). In contrast, categories associated with immune response, cell adhesion, signal transduction, protein refolding, and cell cycle were enriched in the AMA-downregulated dataset (Fig. 2A bottom panel and Table S2). Accordingly, lower levels of transcripts encoding innate and adaptive immune mediators (*Aif1*, *C1rb*, *Ccl5*, *Ifitm3*, *Il18bp*, *Irf7*, *Ly86*, *Lyz1*, *Nfil3*, *Ptger3*, *Tnfrsf14*), regulators of antigen presentation (*Cd74*, *H2-Aa*, *H2-Ab1*, *H2-Eb1*, *Unc93b1*), and adhesion molecules (*Icam1*, *Itgal*, *Itgb7*, *Rac2*) were detected in BMDMs infected with *L. donovani* amastigotes (Fig. 2B). Thus, macrophages undergo widespread changes in the abundance of mRNA subsets associated with downregulation of immune cell functions and upregulation of host cell survival and RNA metabolism upon *L. donovani* amastigote infection.

### Early transcriptional changes in macrophages infected with *L. donovani* promastigotes are indicative of both activation and inhibition of host defense responses

**Figure 3:**
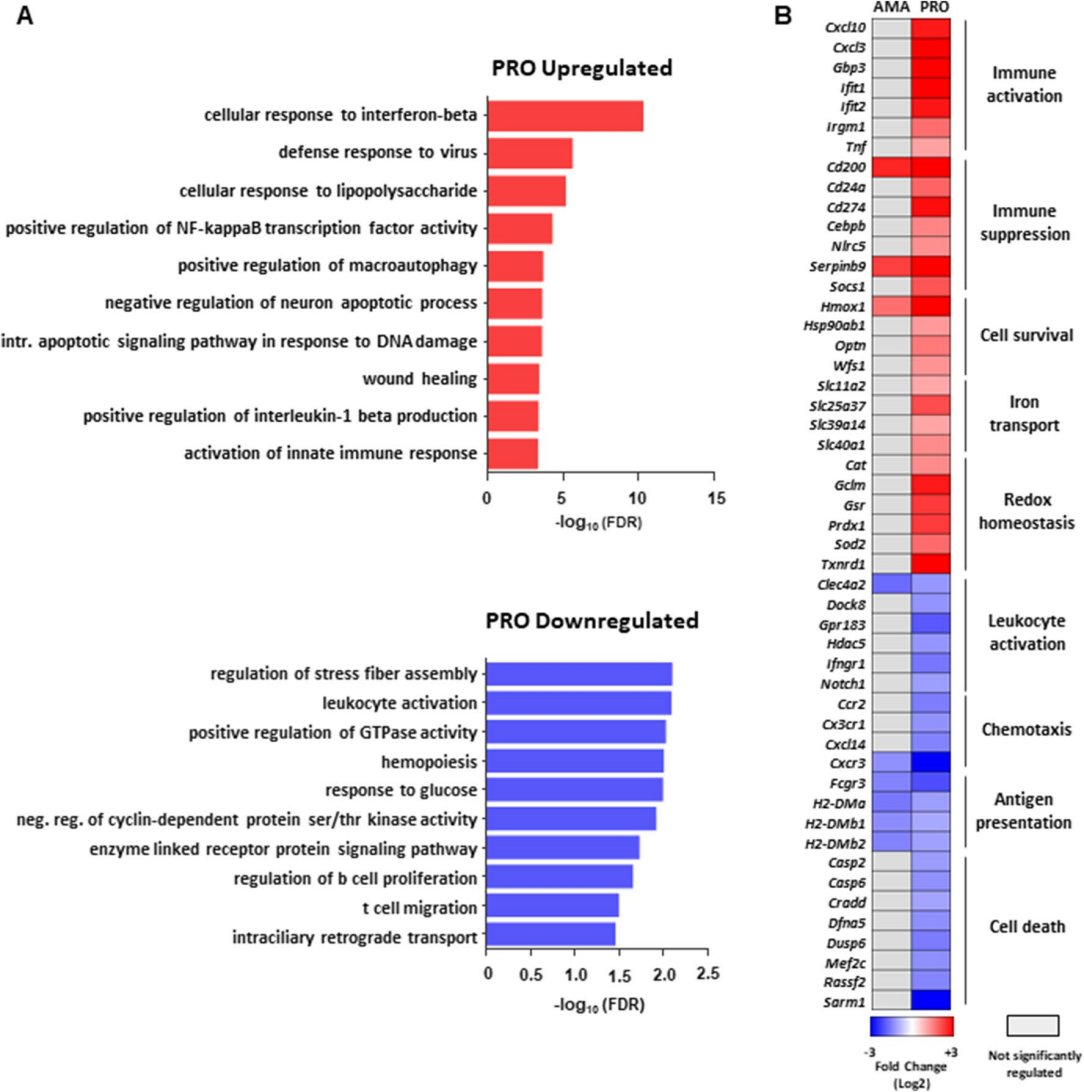
Selective changes in mRNA abundance predict promastigote-specific activation and inhibition of macrophages defense responses during *L. donovani* infection. (**A**) FDR values (− log_10_) for selected GO term enriched categories of differentially up- or downregulated mRNAs upon *L. donovani* PRO infection. (**B**) Changes in mRNA abundance for selected genes in enriched GO terms. Analyses were carried out on data generated from at least three biological replicates.

Enriched GO categories in upregulated transcripts of *L. donovani* promastigote-infected macrophages revealed contrasting groups of activating (i.e. *Cxcl10*, *Cxcl3*, *Gbp3*, *Ifit1*, *Ifit2*, *Irgm1*, *Tnf*) and inhibitory immune factors (*Cd200*, *Cd24a*, *Cd274*, *Cebpb*, *Nlrc5*, *Serpinb9*, *Socs1*) (Fig. 3A upper panel, Fig. 3B and Table S2). In parallel, mRNAs encoding proteins associated with cell survival (*Hmox1*, *Hsp90ab1*, *Optn*, *Wfs1*), iron transport (*Slc11a2*, *Slc25a37*, *Slc39a14*, *Slc40a1*) and redox homeostasis (*Cat*, *Gclm*, *Gsr*, *Prdx1*, *Sod2*, *Txnrd1*) were also overrepresented in the upregulated dataset (Fig. 3A upper panel, Fig. 3B and Table S2). In line with previous observations^6,24^, an increase in the abundance of a group of transcripts associated with lipid metabolism was detected in the PRO data set (*Cd36*, *Lrp12*, *Lpl*, *Acsl1*, *Fabp4*) (Table S1). In contrast, GO categories related to cell death (*Casp2*, *Casp6*, *Cradd*, *Dfna5*, *Dusp6*, *Mef2c*, *Rassf2*, *Sarm1*) and immune functions such as leukocyte activation (*Clec4a2*, *Dock8*, *Gpr183*, *Hdac5*, *Ifngr1*, *Notch1*), chemotaxis (*Ccr2*, *Cx3cr1*, *Cxcl14*, *Cx*cr3), and antigen presentation (*Fcgr3*, *H2-DMa*, *H2-DMb1*, *H2-DMb2*) were enriched in mRNAs with reduced abundance during infection by *L. donovani* promastigotes (Fig. 3A lower panel, Fig. 3B and Table S2). These data indicate that *L. donovani* promastigote infection elicits a transcriptome-wide response in macrophages that results in the upregulation of lipid metabolism, the concomitant expression of activating and inhibitory immune mediators, and the inhibition of cell death and antigen presentation.

### Parasite stage-specific modulation of the host cell transcriptome during *L. donovani* infection

**Figure 4:**
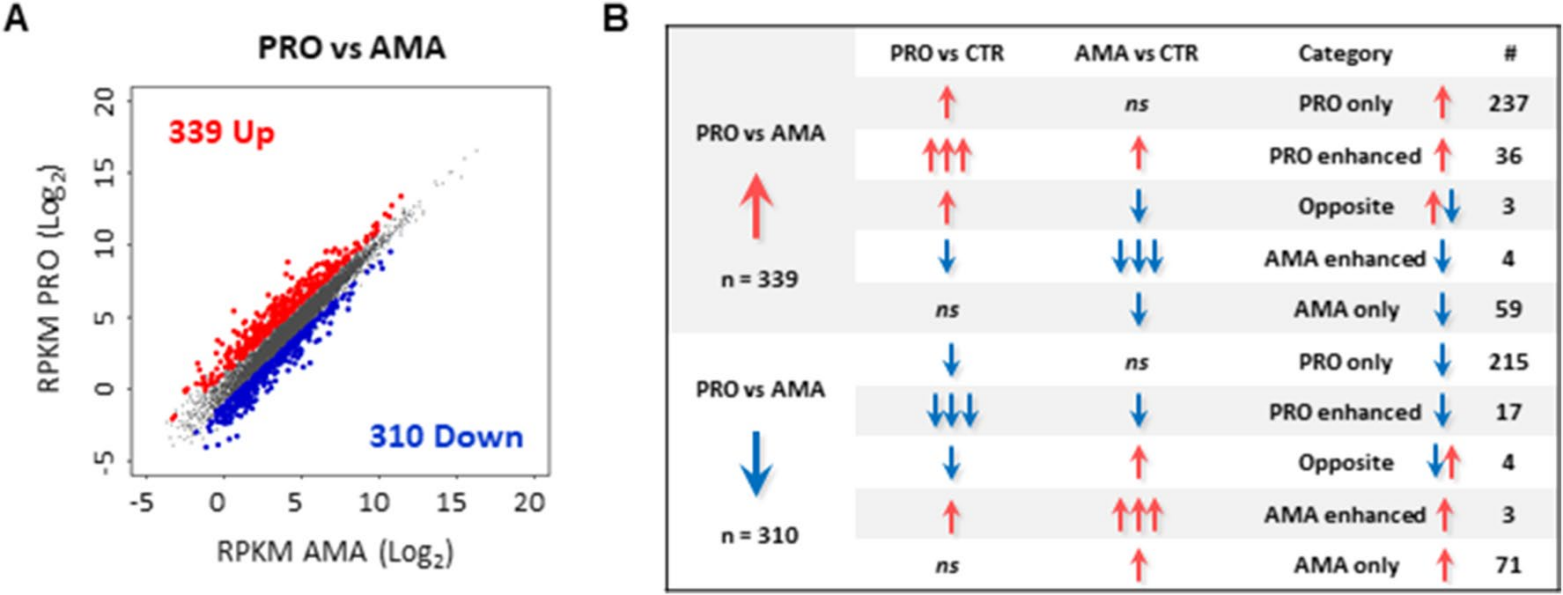
Parasite stage-driven changes in macrophage mRNA abundance during *L. donovani* infection. (**A**) Scatter plot of gene expression as RPKM (log_2_) values for total mRNA between PRO and AMA datasets. Differentially regulated transcripts are indicated in red (upregulated) or blue (downregulated). Unchanged mRNAs are shown in grey. (**B**) Category distribution of transcripts differentially regulated in macrophages upon *L. donovani* PRO versus AMA infection.

**Figure 5:**
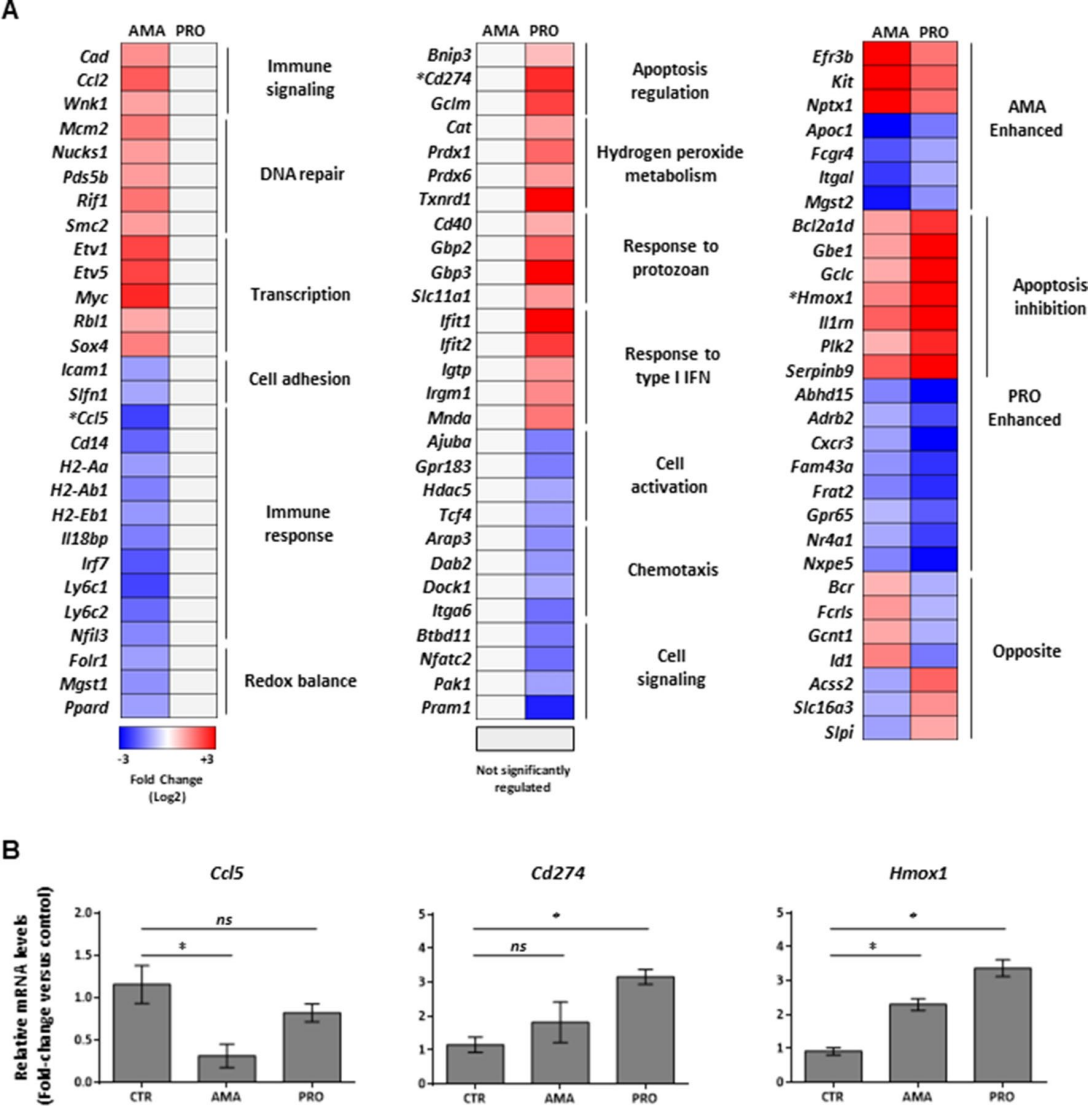
Parasite stage-driven modulation of macrophage transcripts encoding functionally related proteins during *L. donovani* infection*.* (**A**) Heatmaps of selected transcripts differentially regulated only by amastigotes (left panel), promastigotes (middle panel) or both (right panel). Manually curated ontology groups are shown for stage-specific regulated transcripts (left and middle panels). Analyses were carried out on data generated from at least three biological replicates. *Targets selected for validation by RT-qPCR. (**B**) Relative mRNA amounts of *Ccl5*, *Cd274*, and *Hmox1* (normalized to *Actb*) were measured by RT-qPCR. Data are presented as mean ± SD (biological replicates, n = 3). *p < 0.05 (for the indicated comparisons), *ns* non-significant.

Anota2seq identified a subset of mRNAs (n = 649) differentially regulated in the PRO- versus AMA-infected datasets (52.2% upregulated and 47.8% downregulated) (Fig. 4A and Table S1). Comparison of this subset of transcripts with the AMA versus CTRL and PRO versus CTRL contrasts shown in Fig. 1C (Table S1) revealed a complex pattern of regulation with targets exhibiting a stage-exclusive (i.e. PRO only, AMA only), stage-enhanced (i.e. PRO enhanced, AMA enhanced) and stage-opposite (i.e. UP by PRO and DOWN by AMA, DOWN by PRO and UP by AMA) effects (Figs. 4B, 5A and Table S1). In the upregulated PRO versus AMA dataset (n = 339), anota2seq classified 70% of the transcripts as PRO only UP, 17% as AMA only DOWN, and 11% as PRO enhanced UP (i.e. UP by PRO and AMA but with a stronger effect in PRO) (Fig. 4B and Table S1). In the downregulated PRO versus AMA dataset (n = 310), anota2seq classified 69% of the transcripts as PRO only DOWN, 23% as AMA only UP, and 6% as PRO enhanced DOWN (i.e. DOWN by PRO and AMA but with a stronger effect in PRO) (Fig. 4B and Table S1). In addition, 7 transcripts showed an enhanced effect by amastigotes (i.e. AMA enhanced, 3 UP and 4 DOWN) (Figs. 4B, 5A, right panel, and Table S1) whereas 7 transcripts were oppositely regulated between both stages, including 3 that were classified as PRO UP and AMA DOWN (*Acss2*, *Slc16a3*, *Slpi*), and 4 as PRO DOWN and AMA UP (*Bcr*, *Fcrls*, *Gcnt1*, *Id1*) (Figs. 4B, 5A, right panel, and Table S1).

Hierarchical clustering of transcripts identified as exclusively regulated upon amastigote infection (i.e. AMA only) revealed an enrichment of GO categories among upregulated transcripts encoding proteins associated with Immune signaling (*Cad*, *Ccl2*, *Wnk1*), DNA repair (*Mcm2*, *Nucks1*, *Pds5v*, *Rif1*, *Smc2*), Transcription (*Etv1*, *Etv5*, *Myc*, *Rbl1*, *Sox4*), and Cell adhesion (*Icam1*, *Slfn1*) (Fig. 5A, left panel, and Table S2) while downregulated targets exhibited an enrichment of GO categories associated with Immune response (*Ccl5*, *Cd14*, *H2-Aa*, *H2-Ab1*, *H2-Eb1*, *Il18bp*, *Irf7*, *Ly6c1*, *Ly6c2*, *Nfil3*) and Redox balance (*Folr1*, *Mgst1*, *Ppard*) (Fig. 5A, left panel, and Table S2). The same type of analysis in the dataset of exclusively upregulated mRNAs upon promastigote infection (i.e. PRO only UP) identified GO categories related to Apoptosis regulation (i.e. *Bnip3*, *Cd274*, *Gclm*), Hydrogen peroxide metabolism (*Cat*, *Prdx1*, *Prdx6*, *Txnrd1*), Response to protozoan (*Cd40*, *Gbp2*, *Gbp3*, *Slc11a1*), and Response to type I IFN (*Ifit1*, *Ifit2*, *Igtp*, *Irgm1*, *Mnda*) (Fig. 5A, middle panel, and Table S2). By contrast, transcripts exclusively downregulated by promastigotes (i.e. PRO only DOWN) were enriched in GO categories linked to Cell activation (*Ajuba*, *Gpr183*, *Hdac5*, *Tcf4*), Chemotaxis (*Arap3*, *Dab2*, *Dock1*, *Itga6*) and Cell signaling (*Btbd11*, *Nfatc2*, *Pak1*, *Pram1*) (Fig. 5A, middle panel, and Table S2). The PRO enhanced UP subset showed an overrepresentation of apoptosis inhibitors (*Bcl2a1d*, *Gbe1*, *Gclc*, *Hmox1*, *Il1rn*, *Plk2*, *Serpinb9*) (Fig. 5A, right panel, and Table S2). Consistent with this, the activation of a transcriptional regulatory network leading to the inhibition of cell death was identified by Ingenuity Pathway Analysis (IPA) in the PRO upregulated subset (Fig. S1). Unlike the PRO enhanced transcripts, no GO categories were enriched in the AMA enhanced subset (Fig. 5A, right panel). Changes in expression levels of three different transcripts regulated during infection by *L. donovani* amastigotes (*Ccl5*), promastigotes (*Cd274*) or both (*Hmox1*) was confirmed by RT-qPCR experiments (Fig. 5B). Altogether, these results indicate that early infection by amastigotes or promastigotes of *L. donovani* elicits a selective and stage-specific transcriptional signature in macrophages involving mRNAs related to key cellular functions in disease progression.

### Changes in host mRNA abundance upon *L. donovani* infection are associated with a network of upstream transcriptional regulators in macrophages

**Figure 6:**
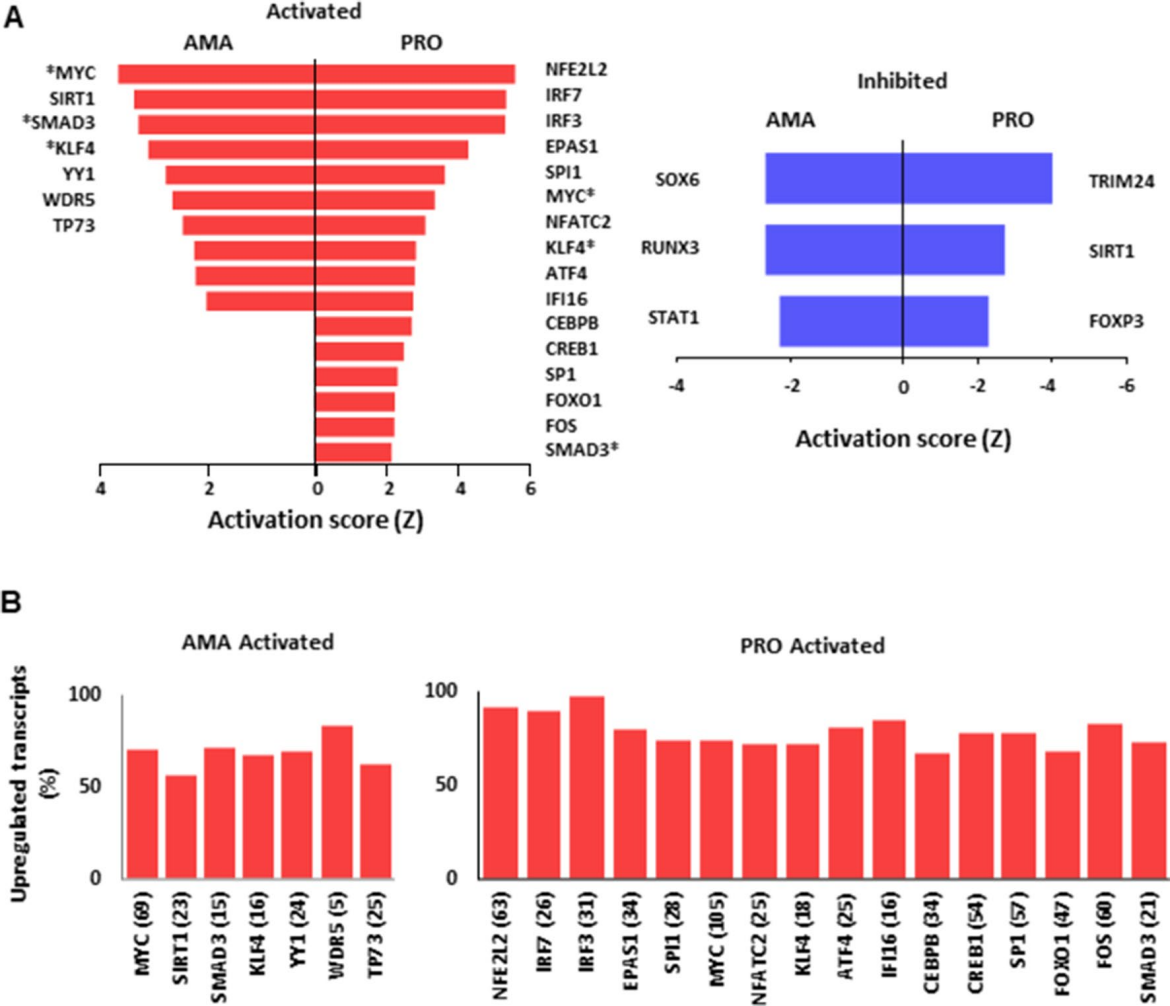
IPA predicts parasite stage-specific modulation of transcriptional regulators in macrophages infected *L. donovani.* (**A**) Activation score (Z) of transcriptional regulators predicted to be involved in the changes of mRNA abundance in macrophages upon *L. donovani* AMA and PRO infection. *Common upstream regulators identified in PRO, AMA datasets by IPA. (**B**) Percentage distribution of upregulated mRNAs associated with upstream transcriptional regulators predicted to be activated in macrophages upon *L. donovani* AMA and PRO infection. Total number of genes regulated by each transcription factor are shown in brackets. Analyses were carried out on data generated from at least three biological replicates.

In order to identify potential upstream regulatory networks responsible for the changes in mRNA levels observed in BMDMs infected by the two life stages of *L. donovani*, we used IPA. With an activation score |Ζ|≥ 2.0 and an FDR ≤ 0.01, IPA identified subsets of transcripts with a regulatory trend predicted to be dependent on the activation or inhibition of different transcriptional modulators in BMDMs infected with *L. donovani* amastigotes or promastigotes (Table S3). Some upstream regulators were common between both parasite stages (MYC, KLF4, and SMAD3) albeit with variations in the number and/or identity of downstream targets in each type of infection (Fig. 6A left panel and Table S3). Others were predicted to be activated only by amastigotes (YY1, WDR5, and TP73) or promastigotes (NFE2L2, IRF7, IRF3, EPAS1, SPI1, NFATC2, ATF4, IFI16, CEBPB, CREB1, SP1, FOXO1, and FOS) (Fig. 6A left panel and Table S3). As expected, transcriptional regulators predicted to be activated upon *L. donovani* infection showed high percentages of associated upregulated mRNAs (Fig. 6B). In agreement with predicted induction of NFE2L2 (a.k.a. NRF2)-dependent transcriptional programs in BMDMs infected with *L. donovani* promastigotes (i.e. 63 genes) (Fig. 6B right panel, Fig. S2A and Table S3), *NRF2-mediated Oxidative Stress Response* was identified by IPA as one of the top networks to be activated by the promastigote stage (Fig. S2B). In addition, a small group of transcription factors was predicted to be inhibited only upon infection with amastigotes (SOX6, RUNX3, and STAT1) or promastigotes (TRIM24, SIRT1, and FOXP3) (Fig. 6A right panel and Table S3). Of note, SIRT1 was predicted to be activated in the amastigote-infected dataset (Fig. 6A left panel and Table S3) whereas the opposite was observed during infection with the promastigote stage (Fig. 6A right panel and Table S3), as previously reported^25^. These data hint at the involvement of a complex regulatory network affecting the abundance of functional subsets of mRNAs in BMDMs infected with *L. donovani* amastigotes or promastigotes.

## Discussion

Early remodelling of the macrophage transcriptome has been reported to be pathogen-specific during bacterial and parasitic infections^17,24,26–28^. Transcriptome-wide analyses of macrophages infected with *L. donovani* have mainly been described at ≥ 12 h post-infection^18,20,21,29–31^, thereby omitting an earlier timeframe during which numerous molecular and cellular changes occurring within infected macrophages^8,9,11,32^ could trigger, or be elicited by, selective reprogramming of the host transcriptome. Herein, using RNAseq, we describe rapid changes in the levels of mRNAs of primary murine macrophages infected with *L. donovani* amastigotes and promastigotes. Distinct transcriptional signatures were identified in macrophages infected with each parasite stage. A marked inhibition of mRNAs encoding proteins related to different immune functions was found in the amastigote-infected dataset whereas a combination of activating and inhibitory immune modulators was observed in promastigote-infected macrophages. Additionally, our in silico analyses identified host mRNA signatures in the up- and downregulated datasets that appear to be under the control of parasite-stage driven networks of transcription factors. These observations indicate that amastigotes and promastigotes of *L. donovani* elicit a complex transcriptome-wide reprogramming in infected macrophages that includes both parasite stage-specific and commonly regulated mRNA subsets.

*Leishmania donovani* amastigote-driven changes in macrophage gene expression have been documented at ≥ 24 h post-infection^18,30,33^. Herein, we provide evidence that *L. donovani* amastigote infection leads to a vast remodelling of the macrophage transcriptome as early as 6 h post-infection. Among the downregulated targets, we found an enrichment in mRNAs encoding proteins related to several macrophage immune functions. IPA predicted that some of these changes are dependent on the inhibition of transcription factor STAT1. In this regard, Matte and Descoteaux previously reported that *L. donovani* amastigotes prevent STAT1 nuclear import and pro-inflammatory gene expression (i.e., *Nos2* and *Irf1*) in BMDMs stimulated with IFNγ^13^. In addition, a transcriptomic study carried out in splenic macrophages revealed that these cells become insensitive to IFNγ during experimental VL despite a strong pro-inflammatory environment in the spleen^30^. Hence, it is plausible that early blockade of STAT1-dependent transcriptional programs in macrophages infected by *L. donovani* amastigotes has a negative effect in IFNγ-mediated microbicidal and immune host responses at later stages of the disease. Further investigation is required to shed light on this matter.

Infection of macrophages results in an oxidative burst response that involves the production of potent microbicidal effectors such as reactive oxygen and nitrogen species^34^. However, the antimicrobial oxidative stress response can also compromise macrophage DNA integrity and lead to the activation of apoptotic signals^35^. Our GO analyses showed an enrichment in mRNAs encoding DNA repair enzymes and inhibitors of apoptosis in the upregulated dataset of *L. donovani* amastigote-infected BMDMs at 6 h post-infection. Similarly, a proteome-based analysis of human macrophages infected with *L. donovani* identified DNA repair as an enriched ontology category reaching maximal values at 24 h post-infection^36^. Moreover, among *L. donovani* promastigote- and amastigote-upregulated transcripts, we detected *Nbn*, which encodes a key member of the MRE11 DNA-damage-sensing complex^37^. Interestingly, *Nbn* is also upregulated in macrophages upon LPS-induced oxidative damage and serves as a modulator of macrophage homeostasis preventing attrition^38^. These reports along with our RNAseq data indicate *L. donovani* amastigotes elicit a cytoprotective transcriptional program to prevent oxidative-driven macrophage apoptosis at early stages of infection. Future studies are necessary to fully understand the molecular underpinnings of parasite-driven activation of the host DNA repair machinery and its role in the establishment and progression of *L. donovani* infection within macrophages.

*Leishmania* parasites inhibit macrophage oxidative burst in order to survive^1^. Recently, Reverte et al*.* showed that expression of the transcription factor NRF2, a master regulator of the antioxidant response^39^, is augmented during *Leishmania* spp. infection, including *L. donovani*^40^. Furthermore, upregulation of NRF2 activity contributed to promote parasite persistence during *L. guyanensis* infection by limiting inflammation^40^. In addition, NRF2-dependent increase in heme oxygenase 1 (HO-1) and ATF3 upon *L. donovani* infection was critical in dampening macrophage oxidative burst and proinflammatory cytokine expression as part of a parasite survival strategy^15^. Thus, our data showing an enrichment of transcripts associated with the activation of an NRF2-dependent antioxidant response in promastigote-infected BMDMs suggest that targeting this regulatory node could be a therapeutic approach to combat VL.

Mounting evidence indicates that specific and abundant changes in the transcriptional landscape of macrophages occur with 1–4 h post-infection with promastigotes of different *Leishmania* species (*L. major*, *L. amazonensis*, *L. chagasi*)^4–7^. For example, microarray data from BMDMs infected with *L. infantum* (syn. *chagasi*) promastigotes for 4 h revealed a marked inhibition of inflammatory transcripts that was concomitant with the upregulation of multiple anti-inflammatory mediators such as TGF-β^7^, a disease severity marker during VL^2^. Even though we did not identify *Tgfb1* in the subset of transcripts upregulated in response to early infection with *L. donovani* promastigotes, we recently described eIF4A-dependent increase in *Tgfb1* mRNA translation efficiency in BMDMs infected with *L. donovani* promastigotes and amastigotes for 6 h^41^. Thus, different VL-causing *Leishmania* spp. (*L. infantum* and *L. donovani*) can lead to similar phenotypes in macrophages, such as rapid production of TGF-β, through different regulatory mechanisms of gene expression.

Our IPA and GO analyses identified a transcriptional signature characterized by early induction of pro- and anti-inflammatory genes in macrophages infected with *L. donovani* promastigotes. These data are in line with previous reports on early reprogramming of the host cell transcriptome by promastigotes of *L. major* and *L. amazonensis*, two *Leishmania* species that cause cutaneous leishmaniasis (CL). A common feature of this type of signature appears to be the upregulation of the pro-inflammatory gene *Tnf* (Fig. 3)^4–6^. TNF levels have been associated with early recruitment of immune cells, including potential host cells, at the site of infection^42^. Thus, it is conceivable that both VL- and CL-causing *Leishmania* species drive rapid *Tnf* transcription and TNF production by macrophages to favor their own replication.

Global-scale profiling of macrophages identified a transcriptional signature associated with the modulation of lipid metabolism during early infection with *L. major* promastigotes^6^. This was further characterized by showing cholesterol accumulation and the dynamics of lipid droplet formation in infected macrophages^24^. Our in silico analyses identified a subset of lipid metabolism-related mRNAs upregulated in the *L. donovani* promastigote-infected data set. Consistent with this, alterations in lipid metabolism have been reported in patients diagnosed with VL^43^. Hence, our data along previous studies indicate that early transcriptional changes triggered by CL- and VL-causing *Leishmania* species contribute to reprogramming lipid metabolism of infected macrophages.

Recently, a transcriptomic analysis of macrophages infected with *L. donovani* promastigotes identified HIF-1α as a negative regulator of the parasite-promoting BNIP3/mTOR/SREBP-1c lipogenesis axis^23^. In parallel, the induction of a transcriptional signature associated with glutamine metabolism was found to be pivotal in VL pathogenesis with a therapeutic potential in synergy with miltefosine treatment^22^. Both studies performed RNAseq on macrophages infected with *L. donovani* promastigotes for 6 h and, although identified transcripts were validated in vivo and in vitro, the global transcriptional response of infected macrophages compared to uninfected controls was not analyzed^22,23^. Even though we did not find an enrichment of HIF-1α-dependent transcripts in our dataset, we detected an increase in *Bnip3*, a transcriptional target of HIF-1α, as previously reported^23^. Similarly, our IPA and GO analyses did not find an enrichment of transcripts associated with glutamine metabolism; however, mRNAs encoding subunits of glutamate-cysteine ligase, a key enzyme in glutathione synthesis and glutamine usage^44^, were upregulated in infected datasets when compared to uninfected controls (i.e. *Gclm* in PRO upregulated, and *Gclc* in PRO and AMA upregulated). In sum, data generated by others and by us indicate that regulation of host cell metabolism is at least in part dependent on parasite-driven transcriptional changes induced by both life stages of *L. donovani* early during infection.

In line with subversion of macrophage immune functions by *L. donovani* promastigotes^1^, we identified a number of mRNAs encoding immune inhibitors in the upregulated promastigote-infected dataset, including *Cd274* (a.k.a. PDL1), *Socs1*, and *Cd200*. PDL1 and its receptor PD1 constitute an important inhibitory axis for T cell activity, and antibody therapy against PD1 has proven successful against numerous malignancies^45^. Notably, the PD1/PDL1 axis was recently identified to play an important role in vivo during VL and immunotherapy against PD1 was effective in hampering parasite burden and pathogenesis^31^. In addition, early induction of SOCS1, a known antagonist of the proinflammatory JAK1/STAT1 pathway^38,46^, was identified as part of a cellular program to prevent oxidative burst-mediated apoptosis in macrophages infected with *L. donovani*^47^. Similarly, a swift increase of CD200 in macrophages exposed to *L. amazonensis* or *L. donovani* infection was described as a strategy to favor parasite proliferation^48–50^. Interestingly, immune blockade of CD200 led to an increase in proinflammatory mediators and parasite elimination capacity of macrophages and T cells, showing its potential as a therapeutic target^49^. Taken together, these reports and our transcriptomic study highlight the early ability of *L. donovani* promastigotes to limit macrophage antimicrobial responses through the modulation of host mRNA abundance.

IPA identified a transcriptional signature associated with type I interferon responses predicted to be activated via the transcription factors IRF3 and IRF7 in the promastigote-upregulated dataset. By contrast, downregulation of *Irf7* mRNA abundance was detected in the transcriptome of amastigote-infected BMDMs. IRF7-dependent parasite elimination was reported in macrophages of the splenic marginal zone during the acute phase of *L. donovani* amastigote infection in vivo (e.g. 5 to 48 h post-infection) and by a cell line of stromal macrophages in vitro*.* Although the expression of IRF7 was not modulated in hepatic macrophages during VL, IRF7-defficient mice showed a decreased ability to control parasite burden in the liver^51^. These observations along with transcriptomic data and our in silico analysis suggest that the ability of macrophages to elicit IRF7-dependent antimicrobial transcriptional programs upon *L. donovani* infection is tissue- and/or parasite-stage specific.

Our group recently described rapid remodeling of the translatome of macrophages infected by promastigotes and amastigotes of *L. donovani*^41^. Herein, we expanded our findings by analyzing early changes in the abundance of host mRNAs during infection. Comparison of the transcriptome and the translatome of *L. donovani*-infected BMDMs at 6 h post-infection indicates that in contrast to changes in translation efficiency^41^, modulation of mRNA abundance is, at least in part, parasite stage-specific. It is plausible that differences in lipid composition^52^ and protein expression^53^ between promastigotes and amastigotes can account for these stage-specific profiles. For example, *L. donovani* promastigotes exhibit a dense glycocalyx comprised of a variety of potent virulence factors (e.g. lipophosphoglycan (LPG), the protease GP63, etc.) that are mostly absent in amastigotes^11,42^. This in turn can affect the process of parasite internalization due to differential usage of macrophage receptors for phagocytosis^54^ leading to distinctive host signaling pathways and transcriptional changes upon infection^1^.

Amastigote-driven changes included the upregulation of transcripts encoding DNA repair modulators while inhibiting those encoding antigen-presenting and macrophage activation factors. Alternatively, promastigote-infected macrophages showed the upregulation of immune inhibitors as well as an antioxidant transcriptional signature associated to NRF2 activity. However, enrichment of transcripts associated with IRF3 and IRF7 suggests that macrophages activate antimicrobial pathways upon *L. donovani* promastigote infection. Interestingly, mRNAs encoding proteins associated with DNA damage-sensing or DNA repair, apoptosis inhibition and mRNA metabolism were upregulated via changes in abundance (Figs. 2, 3 and 4) and translation efficiency^41^. A similar dual effect was observed on a number of downregulated immune-related transcripts (e.g. antigen presentation, leukocyte activation, etc.) (Figs. 2, 3 and 4)^41^. In all, previous studies, along with our current findings support the notion that early parasite-driven changes in macrophage gene expression programs are under the control of transcriptional and post-transcriptional regulatory mechanisms that tailor both protective and harmful host cell responses during *L. donovani* infection.

## Materials and methods

### Reagents and parasites

Culture media and supplements were purchased from Wisent, Gibco, and Sigma-Aldrich. *L. donovani* (LV9 strain) amastigotes were isolated from the spleen of infected female Golden Syrian hamsters (Harlan Laboratories) as previously described^13^. *L. donovani* (LV9 strain) promastigotes were differentiated from freshly isolated amastigotes and were cultured at 26 °C in M199 medium supplemented with FBS (10%), hypoxanthine (100 µM), hemin (5 µM), biopterin (3 µM), biotin (1 µM), penicillin (100 U/mL), and streptomycin (100 μg/mL). Early passage stationary phase promastigotes were used for macrophage infections.

### Ethics statement

Housing and experiments were carried out under protocols approved by the Comité Institutionnel de Protection des Animaux (CIPA) of the INRS—Centre Armand-Frappier Santé Biotechnologie (CIPA 1308-04 and 1710-02). All methods were performed in accordance with relevant guidelines and regulations. These protocols respect procedures on good animal practice provided by the Canadian Council on animal care. The study is reported in accordance with ARRIVE guidelines.

### Differentiation and infection of bone marrow-derived macrophages

Bone marrow-derived macrophages (BMDMs) were differentiated from bone marrow precursor cells isolated from C57BL/6 mice, as previously described^55^. Briefly, marrow was extracted from bones of the hind legs, red blood cells were lysed, and progenitor cells were resuspended in BMDM culture medium supplemented with 15% L929 fibroblast-conditioned culture medium (LCCM). Non-adherent cells were collected the following day and were cultured for 7 days in BMDM culture medium supplemented with 30% LCCM with fresh medium replenishment at day 3 of incubation. BMDMs were then collected, viable cells were counted by trypan blue exclusion and plated in 150 mm petri dishes at a density of 2 × 10^5^ cells/cm^2^ overnight. BMDM cultures were inoculated with *L. donovani* promastigotes or amastigotes at a multiplicity of infection (MOI) of 10:1 for 6 h, as previously described^56^. Glass coverslips were prepared in parallel and stained with HEMA 3 PROTOCOL to assess the rate of infection according to the manufacturer instructions. Promastigote- and amastigote-infected samples averaged at 92.3% ± 2.5% and 86.8% ± 1.9% of infection respectively. Prior to infection, cells were serum-starved for 2 h.

### Cytosolic mRNA extraction

Cytosolic lysates of infected and control BMDMs were prepared for RNA extraction as described^55^. RNA was extracted with QIAzol (QIAGEN) and purified using RNeasy MinElute Cleanup Kit (QIAGEN) according to specifications of the manufacturer. Purity and integrity of RNA was assessed using a Bioanalyzer 2100 with an Eukaryote Total RNA Nano chip (Agilent Technologies).

### RNAseq and data processing

RNAseq libraries were generated using the Smart-seq2 method^57^ and sequenced by using an Illumina HiSeq2500 instrument with a single-end 51-base sequencing setup from three independent biological replicates for uninfected and *L. donovani* promastigote-infected BMDMs, and five independent biological replicates for *L. donovani* amastigote-infected BMDMs. First, RNAseq reads mapping to the reference genome of the Nepalese BPK282A1 strain of *L. donovani* (txid: 981087) were removed (12.7% and 1.4% mappings on average for promastigotes and amastigotes, respectively). The filtered reads were then mapped to the mouse genome assembly GRCm38 (mm10) using HISAT2 with default settings^58^. Gene expression was quantified using the RPKMforgenes.py script^59^ with -fulltranscript -readcount -onlycoding flags from which raw per-gene RNAseq counts were obtained (version last modified 07.02.2014). Genes that had zero counts in all samples were discarded. Annotation of genes was obtained from RefSeq.

### RNAseq data analysis using anota2seq

RNAseq counts were normalized within anota2seq using the default TMM-log2 method^60^. Significant changes in mRNA abundance were identified by anota2seq^60^ using the default parameters with the following modifications: FDR ≤ 0.05; *apvEff* > log_2_(2.0). In anota2seq, the number of contrasts per analysis equals *n*-1 being *n* the number of conditions (i.e. CTR, *Ld* AMA, *Ld* PRO). In analysis one, infections were contrasted to the uninfected control (i.e. *Ld* PRO versus CTR and *Ld* AMA versus CTR); in analysis two, cells infected by different parasite stages were compared together and an additional contrast was included to complete the anota2seq parameters (i.e. *Ld* PRO versus *Ld* AMA and *Ld* PRO versus CTR). Identifiers for genes which cannot be distinguished based on their high sequence similarity (also reported by RPKMforgenes.py), were excluded from downstream analyses.

### Gene ontology analyses

Gene ontology analyses were performed using the PANTHER tool^61^ of the Gene Ontology Consortium (http://geneontology.org/) on the union of transcripts activated or inhibited in BMDMs infected by *L. donovani* amastigotes or promastigotes. Heatmaps of abundance of transcripts activated or inhibited in BMDMs infected by *L. donovani* amastigotes or promastigotes were generated using MORPHEUS. (https://software.broadinstitute.org/morpheus/index.html, Broad Institute).

### Ingenuity pathway analysis

Enrichment of transcripts showing differential abundance in specific functional networks was determined using Ingenuity Pathway analysis (IPA; QIAGEN) by comparing anota2seq-regulated gene sets against the entire sequenced datasets^62^. Within the IPA application, statistical significance was calculated using a right-tailed Fisher Exact test and p-values were adjusted for multiple hypothesis testing using the Benjamini–Hochberg method to arrive at a FDR.

### Quantitative RT-PCR

Purified RNA (500 ng) was reverse transcribed using the LunaScript RT SuperMix Kit (New England Biolabs, cat#E3010L). Quantitative PCR was performed with Luna Universal qPCR Master Mix (New England Biolabs, cat#M3003L). Relative quantification was calculated using the comparative Ct method (ΔΔCt)^63^ and relative expression was normalized to mouse β-actin. Experiments were performed in independent biological replicates (n = 3); each sample was analyzed in a technical triplicate, the average of which was plotted against the respective conditions used. Primers were designed using NCBI Primer-BLAST (http://www.ncbi.nlm.nih.gov/tools/primer-blast/) (Table S4).

## Supplementary Information

Supplementary Figure 1.

Supplementary Figure 2.

Supplementary Table S1.

Supplementary Table S2.

Supplementary Table S3.

Supplementary Table S4.
