# Supplementary material for: Transcriptional profiling of macrophages reveals distinct parasite stage-driven signatures during early infection by Leishmania donovani
Source: Sci Rep. 2022 Apr 16;12:6369. doi: 10.1038/s41598-022-10317-6 (PMC9013368; doi:10.1038/s41598-022-10317-6)
Supplement: Supplementary file 6 — Supplementary Table S4. [file 41598_2022_10317_MOESM6_ESM.pdf]

Table S4. Primer sequences used for RT-qPCR analyses

Gene		Primer sequence (5' - 3')
Actb	Forward	5'- CACCCACACTGTGCCCATCTACGA -3'
	Reverse	5'- CAGCGGAACCGCTCATTGCCAATGG -3'
Ccl5	Forward	5'- TCACCATATGGCTCGGACACC -3'
	Reverse	5'- CACACTTGGCGGTTCCTTCG -3'
Cd274	Forward	5'- CTGAGTTACCCCAGTACCAAGG -3'
	Reverse	5'- AAGTGAGGCGTCTGTGTTTG -3'
Hmox1	Forward	5'- AACCCCAGATCAGCACTAGC -3'
	Reverse	5'- GTTGCCAACAGGAAGCTGAG -3'
